# Increased Serum Levels of Oxytocin in ‘Treatment Resistant Depression in Adolescents (TRDIA)’ Group

**DOI:** 10.1371/journal.pone.0160767

**Published:** 2016-08-18

**Authors:** Tsuyoshi Sasaki, Kenji Hashimoto, Yasunori Oda, Tamaki Ishima, Madoka Yakita, Tsutomu Kurata, Masaru Kunou, Jumpei Takahashi, Yu Kamata, Atsushi Kimura, Tomihisa Niitsu, Hideki Komatsu, Tadashi Hasegawa, Akihiro Shiina, Tasuku Hashimoto, Nobuhisa Kanahara, Eiji Shimizu, Masaomi Iyo

**Affiliations:** 1 Department of Child Psychiatry, Chiba University Hospital, Chiba, Japan; 2 Departments of Psychiatry Chiba University Graduate School of Medicine, Chiba, Japan; 3 Division of Clinical Neuroscience, Chiba University Center for Forensic Mental Health, Chiba, Japan; 4 Division of Medical Treatment and Rehabilitation, Chiba University Center for Forensic Mental Health, Chiba, Japan; 5 Departments of Cognitive Behavioral Physiology, Chiba University Graduate School of Medicine, Chiba, Japan; University of Texas Health Science Center at San Antonio Cancer Therapy and Research Center at Houston, UNITED STATES

## Abstract

**Objective:**

‘Treatment-resistant depression’ is depression that does not respond to an adequate regimen of evidence-based treatment. Treatment-resistant depression frequently becomes chronic. Children with treatment-resistant depression might also develop bipolar disorder (BD). The objective of this study was to determine whether serum levels of oxytocin (OXT) in treatment-resistant depression in adolescents (TRDIA) differ from non-treatment-resistant depression in adolescents (non-TRDIA) or controls. We also investigated the relationships between serum OXT levels and the clinical symptoms, severity, and familial histories of adolescent depressive patients.

**Methods:**

We measured serum OXT levels: TRDIA (n = 10), non-TRDIA (n = 27), and age- and sex- matched, neurotypical controls (n = 25). Patients were evaluated using the Children’s Depression Rating Scale-Revised (CDRS-R) and the Depression Self-Rating Scale for Children-Japanese Version (DSRS-C-J). The patients were also assessed retrospectively using the following variables: familial history of major depressive disorder and BD (1st degree or 2nd degree), history of disruptive mood dysregulation disorder, recurrent depressive disorder (RDD), history of antidepressant activation.

**Results:**

Serum levels of OXT among the TRDIA and non-TRDIA patients and controls differed significantly. Interestingly, the rates of a family history of BD (1st or 2nd degree), RDD and a history of antidepressant activation in our TRDIA group were significantly higher than those of the non-TRDIA group.

**Conclusions:**

Serum levels of OXT may play a role in the pathophysiology of TRDIA.

## Introduction

Major depressive disorder in children and adolescents is characterized by one or more major depressive episodes, defined as at least 2 weeks of persistent change in mood manifested by either a depressed or irritable mood or a loss of interest or pleasure and at least four additional symptoms of depression [1,2]. Depression in children has been reported to be a recurrent and impairing condition associated with increased psychosocial and medical morbidity and mortality [3].

‘Treatment-resistant depression’ is depression that does not respond to an adequate regimen of evidence-based treatment [4]. It is reported that ‘Treatment-resistant depression’ is up to a 50% reduction in depressive symptoms that follows 8–12 weeks of adequate evidence-based treatment [4]. Evidence-based treatments for adolescent depression are selective serotonin reuptake inhibitors (SSRIs), cognitive behavior therapy (CBT), and interpersonal therapy (IPT) [4–6]. However, approx. 40% of depressed adolescent depressive patients who receive evidence-based treatments do not reach remission, so their group is called ‘treatment resistant depression in adolescents (TRDIA)’ [4,7,8]. TRDIA associated with chronic problems which include educational and occupational underattainment, interpersonal problems, alcohol and substance abuse, suicidal behavior, and completed suicide [4,5,7,8]. Children with treatment-resistant depression like ‘TRDIA’ might also develop bipolar disorder or schizophrenia [9]. However, the precise neurobiological mechanisms underlying the pathophysiology of pediatric depression are currently unknown. One way to combat this disorder would be to discover novel biomarkers for it [10].

Recently, we have studied serum oxytocin (OXT) levels in pediatric patients with Attention Deficit/Hyperactivity Disorder (AD/HD) [11]. We found that that decreased levels of OXT may play a role in the pathophysiology of patients with AD/HD and its inherent inattentiveness. Other studies show that OXT is effective in stabilizing mood in humans [12]. Lower plasma OXT levels have been reported in patients with major depression [13]. However, a 2013 study revealed higher serum OXT levels in adult manic-episode patients compared to depressive episode group, remission group and control groups, both before and after response to treatment. These studies suggest that OXT may be a trait marker in bipolar disorder [14].

Based on the above-described findings, we hypothesized that the serum levels of OXT in adolescent patients with treatment-resistant depression are higher than those in adolescents with non-treatment-resistant depression or normal controls, since children with treatment-resistant depression might develop bipolar disorder [9]. Furthermore, a 2012 study supports the importance of understanding the clinical significance of sub-syndromal manic symptoms for the management of treatment resistant depression in adolescents [9]. The objective of this study was to determine whether serum levels of OXT in adolescent patients with treatment-resistant depression differ from those of sex- and age-matched individuals with non-treatment-resistant depression or controls. We also investigated the relationships between serum OXT levels and the clinical symptoms, severity, and familial histories of adolescent depressive patients.

## Methods

### Ethics statement

The ethics committee of the Chiba University Graduate School of Medicine approved the study protocol (IRB no. 137), which was performed in accord with the Declaration of Helsinki II. After receiving a full explanation of the study as well as any potential risks and benefits, all subjects and their parents provided written informed consent for study participation.

### Study design and subjects

Thirty-seven pediatric patients with pediatric depression were recruited from the outpatients of Chiba University Hospital. Twenty-five healthy age- and sex-matched, typically developing control subjects were recruited via advertisements from among the residents of Chiba (city), Japan. All patients were diagnosed by a child psychiatrist according to the ICD-10 criteria [2] for depressive episodes classified as having one of three subtypes (mild-depressive episode subtype, moderate-depressive episode subtype or severe-depressive episode subtype) (Table 1). The 37 patients were also classified into two subtypes: treatment resistant depression in adolescents (TRDIA) subtype (n = 10), and non-treatment resistant depression in adolescent (non-TRDIA) subtype (n = 27).

**Table 1:** Demographic Characterization of Adolescent Patients with TRDIA or non-TRDIA and Normal Controls.

	Adolescent Depression (n = 37)		Control (n = 25)	TRDIA vs. non–TRDIA		TRDIA vs. non–TRDIA vs. HNC	
	TRDIA (n = 10)	non–TRDIA (n = 27)	HNC	t	p	F	p
**Gender (male/female)**	6 / 4	11 / 16	12 / 13	–	–	–	ns^d^
**Age (years)**	14.40 ± 1.71	12.89 ± 1.99	12.88 ± 3.24	–	–	–	ns^b^
**Severity of illness: ICD–10 (Mild/Moderate/Severe)**	(0/4/6)	(10/5/12)	–	–	ns^d^	–	–
**Duration of illness (months)**	23.80 ± 13.94	7.85 ± 6.70	–	–3.474^a^	0.006*^a^	–	–
**CDRS-R Score (range)**	54.40 ± 11.65 (35–64)	51.00 ± 16.68 (26–80)	–	–0.591^a^	ns^a^	–	–
**DSRS-C Score (range)**	22.70 ± 7.30 (8–32)	19.89 ± 7.50 (2–37)	–	–1.023^a^	ns^a^	–	–
**The no. of months followed up after serum sample collection (range)**	46.50 ± 17.70 (24–69)	19.78 ± 20.43 (1–70)	–	–3.652^a^	0.001*^a^	–	–
**No. of RDD**	10	4	–	–	<0.001*^d^	–	–
**No. of past history of DMDD**	5	9	–	–	ns^d^	–	–
**No. of family history of MDD (1**^**st**^ **degree/2**^**nd**^ **degree)**	3 (3/0)	12 (11/1)	0 (0/0)	–	ns^d^	–	–
**No. of family history of BD (1**^**st**^ **degree/2**^**nd**^ **degree)**	4 (3/1)	2(2/0)	0 (0/0)	–	0.035*^d^	–	–
**No. of past history of antidepressant activation**	5	4	–	–	0.041*^d^	–	–
**Oxytocin (pg/mL)**	394.30 ± 371.23	94.34 ± 31.24	117.38 ± 47.33	–	–	–	0.031*^c^

Values are mean ± SD. ns, not significant.

*p<0.05.

^a^ Student's t-test was used to examine the differences between the TRDIA and non-TRDIA groups.

^b^ One-way ANOVA followed by Tukey's multiple comparison was used for comparisons of age in the TRDIA and non-TRDIA groups, and controls.

^c^ For comparisons of the TRDIA and non-TRDIA and healthy normal control (HNC) group, a non-parametric Kruskal Wallis test was used for the comparisons.

^d^ Fisher’s exact test.

BD, bipolar disorder; CDRS-R, Children's Depression Rating Scale-Revised; DMDD, Disruptive Mood Dysregulation Disorder; DSRS-C, Birleson Depression Self-Rating Scale for children, Japanese version; MDD, major depressive disorder; RDD, recurrent depressive disorder; TRDIA, treatment resistant depression in adolescents.

We defined TRDIA as not responding to treatment with two different classes of antidepressants at least for 8 weeks and not responding to at least eight sessions of CBT, following the TRDIA definition of Sakolsky and Smith [15,16]. None of the patients fulfilled the DSM-IV criteria [1] of any of the autism spectrum disorders or AD/HD.

The patients were also assessed retrospectively using the following variables: duration of illness at the time point when the serum sample was collected, familial history of major depressive disorder and bipolar disorder (1st degree/2nd degree), history of disruptive mood dysregulation disorder (DMDD) [17], recurrent depressive disorder (RDD) [2], history of antidepressant activation, and the duration of the period for which we were able to continue to evaluate the patient’s symptoms after the serum sample collection. We clarified 'history of antidepressant activation' manifesting as any combination of the following: irritability, agitation, somatic symptoms of anxiety, panic attacks, restlessness, hostility, aggressiveness, insomnia, disinhibition, emotional labiality, impulsivity, social withdrawal, restlessness, hypomania/mania, paranoia or other psychotic symptoms, or other unusual changes in behavior or mood after antidepressants use following a 2010 study [18] and there is an improvement by the cancellation of antidepressants.

The neurotypical controls (n = 25) underwent a comprehensive medical history assessment to eliminate the individuals with neurological or other medical disorders. The Mini-International Neuropsychiatric Interview for Children and Adolescents (MINI-KID) was also conducted to exclude current and past personal and family histories of mental illnesses [19]. None of the typically developing controls fulfilled any of these exclusion criteria.

### Measurement of clinical symptoms

All patients were assessed using the Children’s Depression Rating Scale-Revised (CDRS-R) and the Depression Self-Rating Scale for Children-Japanese Version (DSRS-C-J). The CDRS-R is a reliable brief rating scale that was designed for 6- to 12-year-olds, and it has been used successfully with adolescents. It is based on a semi-structured interview with the child/adolescent (or an adult informant who knows the child well). The interviewer rates 17 symptom areas (including those that serve as DSM-IV criteria for a diagnosis of depression) [20]. The DSRS-C-J is easy to use and has a predictive value comparable to that of a psychiatric global rating of depressed appearance and history of depression obtained in an interview [21,22]. It was confirmed that the DSRS-C-J can tap an internal dimension of depression and that children are able to evaluate their feeling states with the use of the DSRS-C-J.

### Measurement of OXT levels from serum

Serum samples from the patients and typically developing control subjects were collected between 10:00 and 15:00 h, and stored at −80°C until assayed. Serum OXT levels were measured using the OXT ELISA kit (Catalog no. ADI-900-153, ENZO Life Sciences, Farmingdale, NY). In order to eliminate the effect of potentially interacting molecules, we performed a solid-phase extraction of serum samples. Briefly, an equal volume (250 μL) of 0.1% trifluoroacetic acid in water (TFA-H2O) was added to the serum samples (250 μL) and centrifuged at 17,000 *g* for 15 min, at 4°C. The supernatant was collected. C18 Sep-Pak columns (200 mg, Waters Corp., Milford, MA) were equilibrated with 1 mL of acetonitrile, followed by four applications with 3 mL of 0.1% TFA-H2O.

The supernatant was applied to the C18 Sep-Pak column, and washed four times with 3 mL of 0.1% TFA-H2O, and the flow-through fraction was discarded. Next, the sample was eluted slowly by applying 3 mL of a solution comprising 60% acetonitrile and 40% 0.1% TFA-H2O, and the eluent was collected in a plastic tube. The solvent was evaporated using a centrifugal concentrator under vacuum at 4°C, and the remaining sample was stored at −20°C before the assay.

The samples were reconstituted in assay buffer provided with the ELISA kit, and the assay was performed according to the manufacturer's protocol. Absorbance at 405 nm was then measured using an Emax automated microplate reader (Molecular Devices, Tokyo). All assays were performed in duplicate.

### Statistical analysis

Statistical analyses were performed using the software package SPSS ver. 21.0 for Macintosh (IBM, Armonk, NY) and G*Power3. Fisher’s exact test was used for categorical variables. Student's t-test and the Mann-Whitney U-test were used for the comparisons of continuous variables between pairs of groups. Levene's test was used to determine whether the variables showed equal variance. A one-way analysis of variance (ANOVA) followed by Tukey's multiple comparison was used for comparisons of age in the TRDIA and non-TRDIA groups and the controls. The Kruskal-Wallis test was used for the comparisons of the serum levels of OXT in the TRDIA and non-TRDIA groups and controls. We used Pearson's and Spearman's correlation coefficients and a forced entry multiple regression analysis to detect correlations between serum OXT levels and clinical variables. Statistical significance was set at *p*<0.05 (two-tailed) with power (1-β) = 0.80. The Wilcoxon Mann-Whitney test with 37 total pediatric depression samples and 25 control samples, 10 TRDIA samples, 27 non-TRDIA samples would have enabled us to detect the following effect sizes: total pediatric depression vs. controls, *d* = 0.73 (medium-to-large); TRDIA vs. non-TRDIA, *d* = 1.07 (large); non-TRDIA vs. controls; d = 0.79 (medium to large); TRDIA vs. controls, *d =* 1.08 (large).

## Results

### Sample characteristics

The demographic and clinical characteristics of the TRDIA and non-TRDIA subjects and controls are shown in Table 1. Age and gender did not differ significantly among the three groups. The rates of DMDD and the family history rates of MDD (1st or 2nd degree) did not differ between the non-TRDIA and TRDIA groups. Interestingly, the rates of family history of BD (1st or 2nd degree), RDD and history of antidepressant activation of the TRDIA patients were significantly higher (*p = 0*.*035*, *p<0*.*001*, *p = 0*.*041; respectively*) than those of the non-TRDIA subjects. In addition, the duration of illness in the TRDIA group (23.80 ± 13.94 months) was significantly longer (p = 0.006) than those of the non-TRDIA group (7.85 ± 6.70 months). Furthermore, the number of months of follow-up after the serum sample collection in the TRDIA group (46.50 ± 17.70 months) was significantly longer (p = 0.001) than those of the non-TRDIA group (19.78 ± 20.43 months).

All the ten of the TRDIA patients were receiving drug therapy, as follows.

Patient 1: milnaciplan 100 mg/day and trazodone 50 mg/day

Patient 2: sertraline 25 mg/day, milnaciplan 25 mg/day and trazodone 50 mg/day

Patient 3: milnaciplan125 mg/day, amoxapine 75 mg/day and trazodone 25 mg/day

Patient 4: duloxetine 50 mg/day and quetiapine 75 mg/day

Patient 5: paroxetine 10 mg/day

Patient 6: milnaciplan 75mg/day

Patient 7: fluvoxamine 50mg/day

Patient 8: blonanserine 12 mg/day and lamotrigine 300 mg/day

Patient 9: aripiprazole 3 mg/day

Patient 10: fluvoxamine 50 mg/day and sertraline 25 mg/day

Twenty-three of the non-TRDIA patients were drug-naiïve, and the other four were receiving drug therapy: fluvoxamine (n = 2, 25–100 mg/day), sulpiride (n = 2, 50–100 mg/day). One patient was also diagnosed as having a learning disorder; one patient was diagnosed with a tic disorder; one patient was diagnosed with oppositional defiant disorder, and one patient with learning and oppositional defiant disorders, according to the DSM-IV criteria [1].

### Serum OXT levels

**Fig 1:**
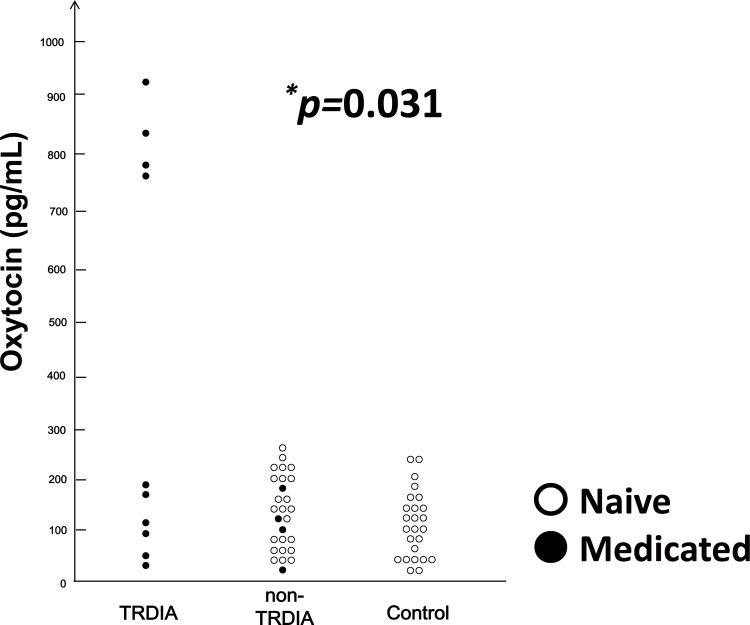
The serum levels of OXT in TRDIA, non-TRDIA and controls. ○ is drug naïve subjects. ● is medicated patients. Kruskal-Wallis test detected significant (*p =* 0.031) differences in OXT serum levels among the TRDIA patients (n = 10), non-TRDIA patients (n = 27) and controls (n = 25). Abbreviations: OXT, Oxytocin; TRDIA, treatment resistant depression in adolescents.

As seen in Table 1 and Fig 1, the Kruskal-Wallis test detected significant (*p =* 0.031) differences in OXT serum levels among the TORDIA patients (394.30 ± 371.23 pg/mL), non-TORDIA patients (94.34±31.24 pg/mL) and controls (117.38±47.33 pg/mL). There was no significant correlation between OXT serum levels and the CDRS-R total scores or DSRS-C-J scores. There were no statistical differences in serum oxytocin levels between the mediated group with adolescent depression and non-medicated group with adolescent depression.

## Discussion

In this study, we found that the serum levels of OXT among the TRDIA and non-TRDIA patients and controls differed significantly. To the best of our knowledge, this is the first report demonstrating differences in serum levels of OXT among TRDIA and non-TRDIA patients and controls.

As noted in the Introduction, an earlier study reported higher serum OXT levels in adult manic-episode patients compared to the depressive episode group, remission group and control groups, both before and after the response to treatment, suggesting that OXT may be a trait marker in bipolar disorder [14]. In the present study, we observed that the serum OXT levels of the TRDIA patients were higher than those of the non-TRDIA patients and controls. Interestingly, the rates of a family history of BD (1st or 2nd degree), RDD and a history of antidepressant activation in our TRDIA group were significantly higher than those of the non-TRDIA group, which may indicate that TRDIA is accompanied by a high risk of the development of bipolar disorder [23].

Considering the meanings of high serum levels of OXT in individuals with TRDIA, we suspect that TRDIA patients with high serum OXT levels might develop bipolar disorder. We also reported that the serum levels of OXT in pediatric patients AD/HD were significantly decreased compared to the levels of neurotypical controls [11]. Thus, serum OXT might be a possible biomarker for differentiating the symptoms of ADHD, bipolar disorders and irritable adolescent depression, because their symptoms are similar regarding irritation, impulsivity and inattentiveness, especially in pediatric to adolescent sufferers.

Lower levels of peripheral OXT have also been identified in some studies of autism spectrum disorders (ASD), depression and schizophrenia, although the findings vary [24–29]. In contrast, a positive correlation was found between OXT levels and symptom severity in patients with social anxiety disorder [30], indicating that excessive attention may raise peripheral OXT levels. From this view point, overactivity and the attention sthenia of bipolar disorder and adolescent irritable depression (which includes TRDIA) may be associated with OXT. In any case, it is necessary to conduct a longitudinal evaluation of these patients, because the diagnosis of pediatric and adolescent patients with mood disorders could be reconsidered.

The main limitation of this study is its small sample size. In addition, we did not compare the serum levels of OXT in ASD and ADHD patients. Further studies using larger sample sizes of other psychiatric disorder cohorts are needed to test our present findings.

Next limitation of this study is evaluation lacking with rating scales about 'history of antidepressant activation' and attachment problems. We did not use the rating scales for 'history of antidepressant activation' and the attachment problems.

Another study limitation is the variation in the follow-up periods after the serum sampling according to the clinical course of each patient. We were also not able to determine whether there were any changes in the diagnoses of the adolescent depression patients in this study (TRDIA and non-TRDIA) over the entire follow-up period. Some of our subjects discontinued their treatment because they achieved a complete recovery. Other participants failed to continue making regular visits, for unknown reasons. We were unable to follow-up the symptoms of the neurotypical controls in this study. Further research with a long-term follow-up study of non-TRDIA and TRDIA patients as well as healthy controls is necessary, especially for subjects with high serum levels of OXT.

## Conclusions

In conclusion, the results of this study suggest that serum levels of OXT may play a role in the pathophysiology of TRDIA, although further research is required to test this concept.

## Supporting Information

S1 File This is the supporting information file of the minimal data set underlying our study. These are minimal excel data of neurotypical controls, non-treatment resistant depression in adolescents (non-TRDIA) and treatment resistant depression in adolescents (TRDIA) < Gender (male/female), Age (years), Severity of illness: ICD-10 (Mild/Moderate/Severe), Duration of illness when serum were collected (months), CDRS-R score, DSRS-C score, The no. of months followed up after serum sample collection, Presence of RDD (recurrent depressive disorder), Presence of past history of DMDD (disruptive mood dysregulation disorder), Presence of familial history of major depressive disorder (1st degree/ 2^nd^ degree), Presence of familial history of bipolar disorder (1st degree/ 2^nd^ degree), Presence of past history of antidepressant activation, Drug therapy when serum sample were collected. Serum levels of Oxytocin (pg/mL)> that readers may contact to request the data, and a confirmation that data will be available upon request to all interested researchers.
(XLSX)
